# Transcutaneous Auricular Vagus Nerve Stimulation Differently Modifies Functional Brain Networks of Subjects With Different Epilepsy Types

**DOI:** 10.3389/fnhum.2022.867563

**Published:** 2022-06-23

**Authors:** Randi von Wrede, Thorsten Rings, Timo Bröhl, Jan Pukropski, Sophia Schach, Christoph Helmstaedter, Klaus Lehnertz

**Affiliations:** ^1^Department of Epileptology, University Hospital Bonn, Bonn, Germany; ^2^Helmholtz-Institute for Radiation and Nuclear Physics, University of Bonn, Bonn, Germany; ^3^Interdisciplinary Center for Complex Systems, University of Bonn, Bonn, Germany

**Keywords:** epileptic brain networks, epilepsy, epilepsy type, transcutaneous vagal nerve stimulation (TVNS), functional networks

## Abstract

Epilepsy types differ by pathophysiology and prognosis. Transcutaneous auricular vagus nerve stimulation (taVNS) is a non-invasive treatment option in epilepsy. Nevertheless, its mode of action and impact on different types of epilepsy are still unknown. We investigated whether short-term taVNS differently affects local and global characteristics of EEG-derived functional brain networks in different types of epilepsy. Thirty subjects (nine with focal epilepsy, 11 with generalized epilepsy, and 10 without epilepsy or seizures) underwent a 3-h continuous EEG-recording (1 h pre-stimulation, 1 h taVNS stimulation, 1 h post-stimulation) from which we derived evolving functional brain networks. We assessed—in a time-resolved manner—important global (topological, robustness, and stability properties) and local (centralities of vertices and edges) network characteristics. Compared to the subjects with focal epilepsies and without epilepsy, those with generalized epilepsies clearly presented with different topological properties of their functional brain network already at rest. Furthermore, subjects with focal and generalized epilepsies reacted differently to the stimulation, expressed as different taVNS-induced immediate and enduring reorganization of global network characteristics. On the local network scale, no discernible spatial pattern could be detected, which points to a rather unspecific and generalized modification of brain activity. Assessing functional brain network characteristics can provide additional information for differentiating between focal and generalized epilepsy. TaVNS-related modifications of global network characteristics clearly differ between epilepsy types. Impact of such a non–pharmaceutical intervention on clinical decision-making in the treatment of different epilepsy types needs to be assessed in future studies.

## Introduction

Epilepsy is one of the most common neurological disorders with a prevalence of 0.5–1% and about 50 million affected subjects (people with epilepsy; PWE) worldwide (GBD 2016 Epilepsy Collaborators, 2019; Hauser and Hesdorffer, 2019; World Health Organization [WHO], 2019). According to the recent proposal of the International League against Epilepsy (ILAE), this disorder is a disease of the brain with at least two unprovoked (or reflex) seizures, or one unprovoked (or reflex) seizure and a probability of at least 60% for further seizures to occur over the next 10 years, or diagnosis of an epilepsy syndrome (Fisher et al., 2014). The most recent ILAE classification of epilepsy provides a very sophisticated schedule for seizure type, epilepsy type and, at each stage of classification, potential etiology of epilepsy (Scheffer et al., 2017). Therefore, the actual classified epilepsy might change over time in some PWE. Nevertheless, classification of epilepsies is substantial for clinical decisions, for clinical and basic epilepsy research as well as for the evaluation and development of new treatment options. Obviously, PWE with structural focal epilepsies might be candidates for epilepsy surgery, PWE with genetic epilepsies due to Glut-1 deficiency are candidates for ketogenic diet, and PWE with limbic encephalitis might profit from immunomodulation. What is more, studies on antiseizure medication (ASM) provide information on efficacy in different epilepsy types, thus providing useful and indispensable information for clinical consultation (Marson et al., 2007a,b, 2021).

The human brain can be understood as a complex network and epilepsy as a network disorder (Bullmore and Sporns, 2009; Berg and Scheffer, 2011). The study of network dynamics can be carried out in spatial as well as in temporal dimensions using different approaches. Electroencephalography is a non-invasive and easy-to-use method in terms of spatial and temporal scales. Tracking network characteristics over time can help to identify intervention-related alterations of brain activity as already been shown by the so-called “pharmaco-EEG” which has provided relevant insights in treatment response, ASM side effects and prediction of those (Höller et al., 2018). Namely, by using an analysis approach that investigates EEG-derived evolving functional brain networks, different global and local network characteristics can be assessed. It is conceivable that different epilepsy types display differences in network characteristics that might provide additional information for differentiating epilepsy types to support clinical evaluation.

ASM is the basis of any epilepsy treatment, but unfortunately for one third of PWE extensive pharmacotherapy attempts have to be undertaken for an at least acceptable seizure situation (Kwan and Brodie, 2000); even the newly developed ASM have not changed this situation significantly (Chen et al., 2018). Pharmacotherapy-resistance is a great burden for PWE and their caregivers. Thus, there is a strong need for alternative or complementary non-pharmaceutical treatment options. Brain stimulation techniques are well established in the treatment of epilepsy. Invasive vagus nerve stimulation (iVNS) is used for decades with more than 100,000 implanted systems (Fisher et al., 2020), and efficacy and safety are well documented over the years with responder (PWE in whom seizure frequency is reduced by more than 50%) rates of up to 50% (Elliott et al., 2011; Morris et al., 2013). Though generally well tolerated and even having a positive impact on mood, risk of anesthesia and surgery have to be considered with an overall complication rate of up to 12%, and surgical complication rate amounts up to 8.6% (Révész et al., 2016). Transcutaneous auricular vagus nerve stimulation (taVNS), the non-invasive external stimulation of the auricular branch of the vagus nerve, is an alternative worth of investigation. Good efficacy, tolerability and usability was previously shown for taVNS (Stefan et al., 2012; Bauer et al., 2016; Barbella et al., 2018; Liu et al., 2018; von Wrede et al., 2019). Most clinical trials have been conducted with PWE with focal epilepsy or in groups consisting of subjects with focal or generalized epilepsy. However, a thorough work up on differences in terms of efficacy in different epilepsy types is missing (Lampros et al., 2021). As the number of participants in above mentioned studies is quite low and only few data from randomized controlled trials is available, a final assessment of the efficacy is not yet available.

To date, the mode of action of vagus nerve stimulation is not fully understood, but may involve alterations of different metabolic pathways (for an overview see Farmer et al., 2021). Hence, it is supposed that VNS leads to a rather unspecific, global activation of various brain structures [including thalamus, limbic system, insular cortex (Rutecki, 1990; Ben-Menachem, 2002)]. Recently, modifications of brain network topology as well as modification of network stability and robustness were shown in a larger group of subjects with and without central nervous system diseases corroborating the idea of an unspecific global activation (Rings et al., 2021; von Wrede et al., 2021). As epilepsy types differ clinically and pathophysiologically, we hypothesized that effects of non-pharmaceutical interventions on functional brain networks in different epilepsy types differ as well. To test this hypothesis, we investigated short-term taVNS-induced immediate and enduring modifications of global and local characteristics of evolving functional brain networks in subjects with different types of epilepsy and non-epilepsy subjects.

## Materials and Methods

### Subjects

Subjects who were admitted to our ward from March 2020 to February 2021 were screened for suitability for this study. Inclusion criteria were clinical necessity (differential diagnosis or electrophysiological follow-up) for long-term video-EEG-recording and age 18 years and older. Exclusion criteria were previous brain surgery, actual or previous neurostimulation such as invasive or non-invasive vagus nerve stimulation or deep brain stimulation, progressive disease, seizures occurring within 24 h before the start of the study, insufficient German language capability, mental disability and incompetence to follow instructions. Demographic data were derived from patient reports, and epilepsy type was classified according to Scheffer et al. (2017). Subjects were assigned to three different groups: focal epilepsy group (G1), generalized epilepsy group (G2), and non-epilepsy group (G3). After being provided with written information and being given the opportunity to ask further questions, 35 subjects volunteered to participate and signed informed consent.

### Transcutaneous Auricular Vagus Nerve Stimulation and Examination Schedule

Following previous studies (Rings et al., 2021; von Wrede et al., 2021), we applied transcutaneous auricular vagus nerve stimulation for 1 h in the early afternoon while the subjects underwent a 3 h continuous video-EEG-recording [1 h pre-stimulation baseline 1 (B1), 1 h taVNS (S) and 1 h post-stimulation baseline 2 (B2)]. During this 3-h block, subjects continued laid-back activities (awake, no other activation methods applied). Stimulation was carried out unilaterally (left cymba conchae) using two hemispheric titanium electrodes of a taVNS device (tVNS Technologies GmbH, Erlangen, Germany) with a set of non-adjustable parameters (biphasic signal form, impulse frequency 25 Hz, impulse duration 20 s, impulse pause 30 s) and individually adjusted intensity of stimulation until the subject experienced a “tingling,” but no painful sensations. All subjects were under stable CNS medication (if taking any) and no activation methods (such as hyperventilation or sleep deprivation) were applied at least 24 h before start of the examination. In order to track possible changes of cognition and behavior, a standardized neuropsychological assessment [EpiTrack^®^ and a modified version of the Adverse Events Profile (AEP)] preceded and followed the EEG-recording. After stimulation the subjects answered a questionnaire on the evaluation of the device usability and tolerability (for details of tests see Supplementary Appendix A).

### Electroencephalogram Recordings and Data Pre-processing

We recorded electroencephalograms (EEG) from 19 electrodes (18 electrode sites according to the 10–20 system and Cz served as physical reference). EEG data were sampled at 256 Hz using a 16 bit analog-to-digital converter and were band-pass filtered offline between 1 and 45 Hz (4th order Butterworth characteristic). To suppress contributions at the line frequency (50 Hz) a notch filter (3rd order) was applied. All recordings were visually inspected for strong artifacts (subject movements, amplifier saturation, or stimulation artifacts) and such data were excluded from further analyses.

### Characterizing Functional Brain Networks on Global and Local Scale

Functional networks consist of vertices and edges. We here associated network vertices with brain regions sampled by the EEG electrode contacts and network edges with time-varying estimates of the strength of interactions between the dynamics of pairs of those brain regions, regardless of their anatomical connections. Following previous studies, we derived evolving, fully connected and weighted networks from a time-resolved synchronization analysis of the above mentioned 3-h EEG-recording, assessed important global and local characteristics of the networks, and tracked their changes over time (for details see Supplementary Appendix B).

On the global network scale, we assessed the topological characteristics average clustering coefficient *C* and average shortest path length *L*. The average clustering coefficient *C* characterizes the network’s functional segregation; the lower *C*, the more segregated is the weighted fully connected network. The average shortest path length *L* characterizes the network’s functional integration; the lower *L*, the more integrated is the weighted fully connected network. In this model, functional segregation (integration) reflects independent (dependent) information processes between brain regions (Tononi et al., 1994).

Furthermore, we assessed the network’s robustness and stability characteristics. Assortativity *A* reflects the tendency of edges to connect vertices with similar or equal properties. If edges preferentially connect vertices with dissimilar properties, such networks are called disassortative. Disassortative networks are more vulnerable to perturbations and appear to be easier to synchronize than assortative networks. Synchronisability *S* assesses the network’s propensity (or vulnerability) to get synchronized by an admissible input activation: the higher *S*, the more easily can the synchronized state be perturbed.

On the local network scale, we assessed importance of vertices and edges using two different and opposing centrality concepts: a path-based and an interaction-strength-based one. Both of them provide non-redundant information about the role vertices and edges play in the larger network. As path-based centrality index, we employed betweenness centrality *C^B^*. A vertex/edge with high *C^B^* is central since it connects different regions of the network as a bridge. As interaction-strength-based centrality index, we employed eigenvector centrality *C^E^*. A vertex/edge with high *C^E^* is central since the vertices/edges connected to it are central as well, therefore it reflects the influence of the vertex/edge on the network as a whole (for details see Supplementary Appendix C).

### Statistical Analyses

For each phase of the examination schedule (B1, S, and B2), we investigated whether the three subject groups (G1, G2, and G3) presented with different global and local network characteristics (Mann-Whitney *U*-test; *p* < 0.05). For each subject group, we investigated whether global and local network characteristics differed between the phases of the examination schedule (Mann-Whitney *U*-test; *p* < 0.05). In addition, and in order to distinguish cases that responded to the stimulation from non-responding cases, we repeated the latter analysis on a single subject level. All *p*-values were corrected for multiple comparisons using the Bonferroni method. Differences in taVNS intensities were investigated in the three subject groups (Kruskal-Wallis test; *p* < 0.05). Eventually, we tested for differences between neuropsychological variables assessed prior to and after the EEG-recording [repeated measures ANOVA; within-subject factor: EpiTrack^®^ pre/post score; between-subject factor: group (G1, G2, and G3); *p* < 0.05]. Furthermore, we investigated whether the assessment of usability of the device differs between the three subject groups (G1, G2, and G3) (Mann-Whitney *U*-test; *p* < 0.05).

## Results

From the thirty-five eligible subjects, five subjects had to be excluded due to EEG data quality. Data from thirty subjects (20 females; age 18–55 years, median 26.5 years) were included in the analyses. Twenty subjects suffered from epilepsy, 9 subjects from focal (G1: 5 females; age 18–55 years, median 26 years) and 11 subjects from generalized epilepsy (G2: 7 females; age 18–54 years, median 22 years). Fifteen of those 20 PWE (75%) had to be considered as drug-resistant according to the definition of the ILAE (Kwan et al., 2010), with 6 PWE with focal epilepsy and 9 PWE with generalized epilepsy. Ten subjects did not suffer from epilepsy and had never experienced seizures before (G3: 8 females; age 19–42 years, median 27.5 years). TaVNS stimulation intensities did not differ significantly between subject groups (G1: range: 0.9–3.5 mA, mean 2.5, *SD* ± 0.8; G2: range: 0.5–3.2 mA, mean 1.6, *SD* ± 0.9; G3: range: 1.0–5.0 mA, mean 2.3, *SD* ± 1.2).

### Global Network Characteristics in Different Epilepsy Groups (G1 and G2) and Non-epilepsy Group (G3)

**FIGURE 1:**
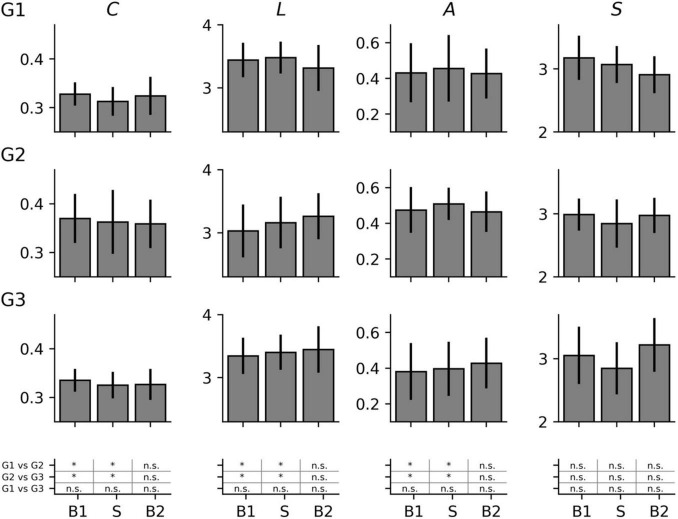
Global network characteristics (average clustering coefficient *C*, average shortest path length *L*, synchronisability *S*, and assortativity *A*) of the investigated groups (G1 = focal epilepsy group, G2 = generalized epilepsy group, G3 = non-epilepsy group) in the three phases of the study (B1 = pre-stimulation baseline 1, S = stimulation, B2 = post-stimulation baseline 2). Mean values and standard deviation. *significant (Mann-Whitney *U*-test, *p* < 0.05), n.s. = non-significant.

On the global network scale (see Figure 1), the focal epilepsy group (G1) and the non-epilepsy group (G3) presented with comparable topological network characteristics (average clustering coefficient *C* and average shortest path length *L*) during all phases of the examination schedule. Contrary to this, we observed the group of subjects with generalized epilepsies (G2) to possess topological characteristics that differed significantly from the characteristics seen in both the focal epilepsy group and the non-epilepsy group. Already before (phase B1) but also during stimulation (phase S), the networks of group G2 were less segregated (higher average clustering coefficient *C*) and more integrated (lower average shortest path length *L*) than the networks of groups G1 and G3. Interestingly, the vanishing differences seen after the stimulation (phase B2) possibly point to a taVNS-mediated topology-modifying effect in the group of subjects with generalized epilepsies. As regards the networks’ stability and robustness characteristics (synchronisability *S* and assortativity *A*), the three subject groups presented with comparable findings during all phases of the examination schedule.

**FIGURE 2:**
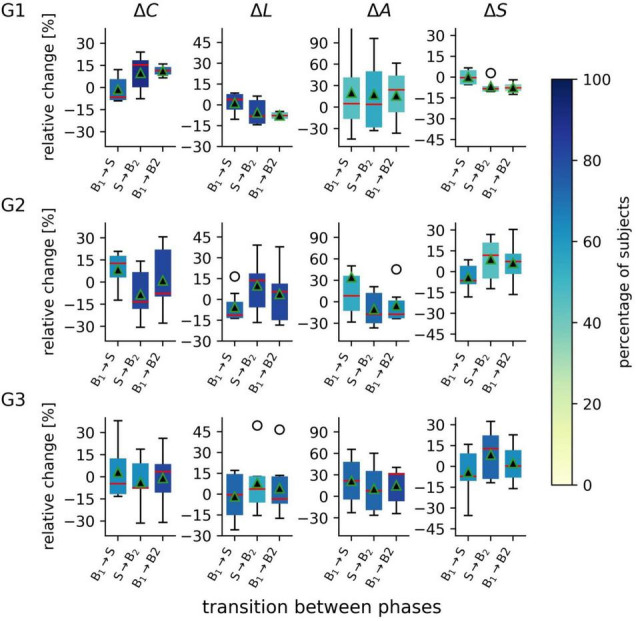
Distributions of taVNS-related alterations in global network characteristics of the three investigated groups (G1 = focal epilepsy group, G2 = generalized epilepsy group, G3 = non-epilepsy group) between the three investigated phases (B1 = pre-stimulation baseline 1, S = stimulation, B2 = post-stimulation baseline 2). Boxplots of relative changes in network characteristics (average clustering coefficient *C*, average shortest path length *L*, synchronisability *S*, and assortativity *A*). Bottom and top of a box are the first and third quartiles, and the red band and the black triangle are the median and the mean of the distribution. The ends of the whiskers represent the interquartile range of the data. Outliers are marked by an o-sign. Boxes are color coded according to the percentage of subjects for whom significant changes in global network characteristics were observed on per subject base.

Testing for differences between network characteristics from each phase led to non-significant results in each subject group (data not shown). On this population sample level, taVNS thus appeared to not immediately affect the investigated global network characteristics. Nevertheless, since not all subjects may display taVNS-mediated changes of their functional brain network (Rings et al., 2021; von Wrede et al., 2021), we specifically investigated those subjects for whom we identified significant changes of their network characteristics (see Figure 2) and observed the subject groups to present with a different pattern of change. When the networks of both the focal epilepsy group (G1) and the non-epilepsy group (G3) transited from phase B1 to phase S, their average clustering coefficient *C* decreased (relative change of median values in G1: −6.5%; G3: −4.7%) while the average shortest path length *L* of G1 increased (+ 3.9%) and changes were negligible for G3 (−0.4%). This points to an immediate stimulation effect that renders these networks more segregated and, at least for G1 less integrated. When comparing network characteristics from the phases prior to (B1) and after the stimulation (B2), we could identify an enduring effect that rendered network less segregated (*C* increased; G1: + 11.4%, G3: + 3.6%) and more integrated (*L* decreased; G1: −7.8%; G3: −3.5%). Interestingly, for the networks of the generalized epilepsy group (G2), we observed these stimulation-mediated changes to present with an inverted pattern: the immediate stimulation effect resulted in less segregated (*C* increased by + 12.8%) and more integrated networks (*L* decreased by −11.3%), while the enduring effect presented with more segregated (*C* decreased; −7.6%) and less integrated networks (*L* increased; + 5.7%).

TaVNS exerted an immediate robustness-enhancing effect over the networks in all groups (changes in assortativity *A*; G1: + 4.9%; G2: + 8.4%; G3: + 21.8%). On the longer term (comparing phases B1 and B2), we observed a strong robustness-enhancing enduring effect for the focal epilepsy group and the non-epilepsy group (G1: + 24.4%; G3: 31.4%). In contrast, in the generalized epilepsy group appeared to have a robustness-decreasing enduring effect (G2: -17.4%).

As regards network stability, we observed taVNS to decrease the networks’ vulnerability of the synchronized state to get perturbed when transiting from phase B1 to phase S in the generalized epilepsy group and the non-epilepsy groups (changes in synchronisability *S*: G2: −6.4%; G3: −7.3%) while this immediate effect in the focal epilepsy group was negligible (G1: −0.4%). Interestingly, in the focal epilepsy group this minor reduction increased into the post-stimulation phase (G1: −7.6%), while taVNS had an enduring vulnerability-enhancing effect on the networks in the generalized group (G2: + 7.4) and a negligible effect in the non-epilepsy group (G3: + 0.3%).

### Local Network Characteristics in Different Epilepsy Groups (G1 and G2) and Non-epilepsy Group (G3)

**FIGURE 3:**
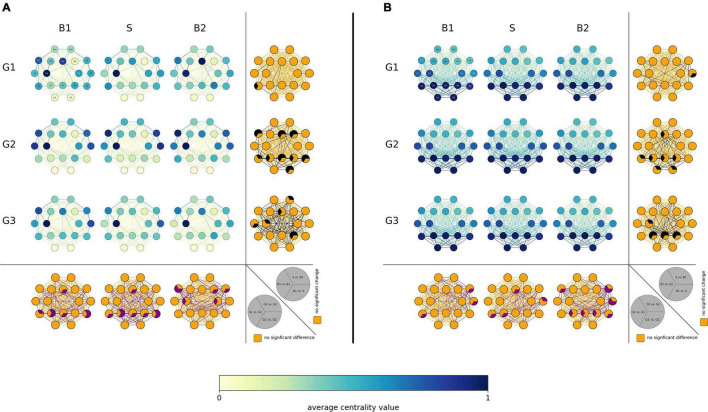
Local network characteristics. [**(A)** Betweenness centrality *C^B^* and **(B)** eigenvector centrality *C^E^*] of the three investigated groups (G1 = focal epilepsy group, G2 = generalized epilepsy group, G3 = non-epilepsy group) in the three investigated phases (B1 = pre-stimulation baseline 1, S = stimulation, B2 = post-stimulation baseline 2). Network vertices arranged according the international 10–20 system for EEG-recording (electrode naming see first plot). Color coding of vertices and edges according to the average centrality values. Bottom: Difference between groups (G1, G2, G3) for local network characteristics in the three investigated phases. Orange: no significance, purple: significant difference (*p* < 0.05). Right side each plot: Differences between phases (B1, S, B2) for local network characteristics in the three investigated groups. Orange: no significance, black: significant change (*p* < 0.05).

On the local network scale (see Figure 3), we obtained different results on the population sample level depending on the employed vertex centrality concept. Betweenness centrality highlighted vertices associated with left fronto-centro-temporal brain regions as most important (high *C^B^* values) in all subject groups. In contrast, eigenvector centrality highlighted a posterior-anterior gradient of vertex importance with the most important (high *C^E^* values) vertices associated with posterior brain regions in all subject groups. Most important vertices differed significantly neither between groups nor between phases, apart from some few, locally mostly unspecific differences seen particularly for the generalized and non-epilepsy group. As regards the importance of edges, i.e., of interactions between brain regions, none of the employed edge centrality concepts highlighted a clear-cut spatial pattern of differences, neither between groups nor between phases. On the population sample level, taVNS thus appeared to have an only minor (if at all) immediate impact on the investigated local network characteristics.

**FIGURE 4:**
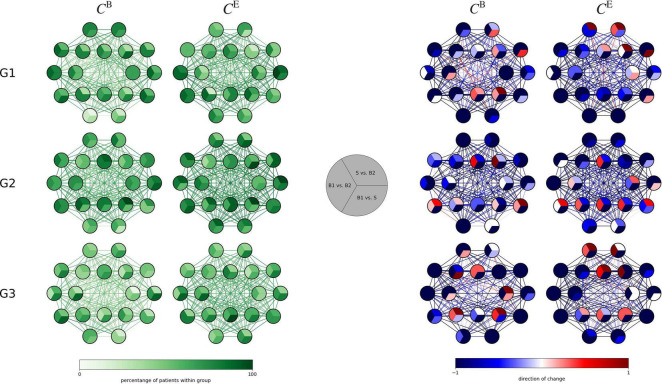
**(Left)** Distributions of taVNS-related alterations in local network characteristics (betweenness centrality *C^B^* and eigenvector centrality *C^E^*) of the three investigated groups (G1 = focal epilepsy group, G2 = generalized epilepsy group, G3 = non-epilepsy group) between the three investigated phases (B1 = pre-stimulation baseline 1, S = stimulation, B2 = post-stimulation baseline 2). Network vertices arranged according the international 10–20 system for EEG-recording. Color coding of vertices and edges according to the percentage of subjects for whom significant changes in local network characteristics were observed on per subject base. **(Right)** Differences between phases (B1, S B2) for local network characteristics in the three investigated groups. Color coding of vertices and edges according to the direction of change of importance for vertices and edges (per subject base).

Proceeding as above and investigating solely those subjects that presented with significant taVNS-mediated changes of their local network characteristics, we observed that most subjects displayed such changes (see Figure 4). Interestingly, the highest proportion of subjects showing significant changes was seen in the generalized epilepsy group, however, with no discernible spatial pattern of change. In contrast, for most subjects from the focal epilepsy group, taVNS-mediated changes of vertex importance (assessed with betweenness centrality) were confined to vertices associated with fronto-temporal brain areas. Other taVNS-mediated alterations of vertex or edge centralities presented as diffuse with no clear-cut spatial pattern.

### Stimulation-Related Change of EpiTrack^®^ Score and Subjective Measures

An improvement in attentional-executive functioning as measured with EpiTrack^®^ from pre- to post-assessment (main effect time: *F* = 28.97, *p* < 0.001), but no interaction effect of time and group (*F* = 1.31, *p* = 0.29) was observed. Seven subjects (23.3%; 2 in G1, 1 in G2, 4 in G3) showed a significant intraindividual improvement (EpiTrack^®^; ≥ 4 points). None of the subjects worsened significantly. No significant self-perceived changes were observed regarding the total scores in the cognitive, behavioral, and physiological domains of the modified Adverse Events Profile (*p* > 0.05).

#### Usability, Tolerability and Side Effects of Transcutaneous Auricular Vagus Nerve Stimulation

Usability data were analyzed across all subjects as there were no differences between groups (*p* > 0.05). Handling of the device was rated as good or very good by all subjects. 93.1% felt that the continuation of their activities was not affected by the stimulation. Wearing comfort was rated as good or very good by 83.3% of the subjects. Most subjects stated that the device is well or very well suited for long-term use during the day (80%) or repeated use within 1 day (86.6%). Side effects were neither reported nor clinically observed.

## Discussion

In this study, we investigated whether global and local characteristics of functional brain networks differ between different types of epilepsy and non-epilepsy subjects and whether short-term taVNS differently modifies their global and local network characteristics. In the following, we discuss our findings in the light of the available research results.

### Global and Local Network Characteristics Differ Between Different Epilepsy Types During Rest Phase

We observed significant differences between global characteristics (average clustering coefficient and average shortest path length) of networks from subjects with generalized epilepsies, focal epilepsies and from non-epilepsy subjects, which corroborates previous studies (Niso et al., 2015; Drenthen et al., 2020). Here, subjects with generalized epilepsies presented with less segregated and more integrated functional brain networks. These findings are in line with earlier studies (Chavez et al., 2010; Chowdhury et al., 2014), though contrast with another study (Zhang et al., 2011). Network studies in epilepsy and especially epilepsy syndromes is an evolving research field, and although results and knowledge are published at a tremendous pace, the applied methods differ and results are not easy to reconcile and might therefore explain opposing results. On the local scale and in par with previous studies (Lohmann et al., 2010; van den Heuvel and Sporns, 2013; Rings et al., 2021), different brain regions were highlighted as important with the different centrality concepts. Our findings for the three subject groups, namely left fronto-central brain regions are characterized as most important with betweenness centrality and parieto-occipital regions with eigenvector centrality, are in line with previous observations (Lohmann et al., 2010; van den Heuvel and Sporns, 2013). Differences in functional connections between brain regions were negligible as no clear-cut spatial differences were observed between subjects with generalized epilepsies, focal epilepsies and non-epilepsy subjects.

Summarizing these findings, we could show that already during rest global but not local characteristics of functional brain networks are different in generalized epilepsies compared to focal epilepsies and the non-epilepsy group. Results derived from brain network analyses might thus provide additional information for differentiating between different types of epilepsy, and thereby supporting a thorough work-up for classification of epilepsy type which is indispensable for optimal patients’ care.

### Transcutaneous Auricular Vagus Nerve Stimulation Differently Modifies Global and Local Network Characteristics in Different Epilepsy Types

As in previous studies (Redgrave et al., 2018; von Wrede et al., 2021), taVNS was easy to use, well tolerated and without negative impact on attention and executive function; in some subjects these even improved.

On the global network scale, short-term taVNS induced modifications of topology-, robustness-, and stability-associated network characteristics in the majority of investigated subjects as it was observed in previous studies (Rings et al., 2021; von Wrede et al., 2021). A taVNS-related enduring topological reorganization of functional brain networks in focal epilepsies in terms of a more integrated and less segregated network structure was shown recently (von Wrede et al., 2021). Extending this finding, we here observed modifications of functional brain network organization to differ between different epilepsy types. We found an inverted pattern of reorganization between focal and generalized epilepsies, with the latter displaying an immediate reorganization toward a more integrated/less segregated and an enduring reorganization toward a more segregated/less integrated network. The taVNS-mediated topological reorganization of functional brain networks in the non-epilepsy subjects resembled those of the focal epilepsy group though being less pronounced. These epilepsy-type-related findings might explain the differing results for immediate modifications of brain network reorganization by taVNS reported previously (Rings et al., 2021).

TaVNS induced a comparable immediate robustness-enhancing modification of the functional brain networks of subjects with focal and generalized epilepsies as well as non-epilepsy subjects. The enduring effect, however, clearly differed between epilepsy types: robustness increased in the focal epilepsy group (which is in par with a previous study (von Wrede et al., 2021), but decreased in the generalized epilepsy group. What is more, taVNS induced an enduring higher vulnerability for perturbation in generalized epilepsies and a lower one in focal epilepsies, leading to different network stability.

Interestingly, on a local network scale, more subjects with generalized epilepsy than with focal epilepsy displayed taVNS-induced modifications of importance of brain regions and functional connections. We hypothesize that in focal epilepsies important brain regions are more susceptible for modifications, whereas in generalized epilepsy the pattern of modified brain regions is more diffuse. No clear-cut spatial pattern could be observed for the importance of functional connections.

Summarizing these findings (see Table 1), we could provide first evidence that in subjects with generalized or focal epilepsy, short-term taVNS differently modified global characteristics of their functional brain networks. Local network characteristics remained largely unaffected as already reported on previously (Rings et al., 2021).

**TABLE 1:** Synopsis of taVNS-induced immediate and enduring modifications of global and local characteristics of weighted fully connected functional brain networks in different epilepsy types.

		Focal epilepsy group	Generalized epilepsy group	Non-epilepsy group
**Global network scale**				
Topology	Immediate effect	Segregation **↑** integration ↓	Segregation ↓ integration ↑	Segregation ↑ integration ↔
	Enduring effect	Segregation ↓ integration ↑	Segregation ↑ integration ↓	Segregation ↓ integration ↑
Robustness	Immediate effect	↑	↑	↑↑
	Enduring effect	↑↑	↓↓	↑↑
Stability of the synchronized state	Immediate effect	↔	↑	↑
	Enduring effect	↑	↓	↔
**Local network scale**				
Path-based centrality index	Vertices	Diffuse	Diffuse	Diffuse
	Edges	Diffuse	Diffuse	Diffuse
Interaction-strength-based centrality index	Vertices	Diffuse	Diffuse	Diffuse
	Edges	Diffuse	Diffuse	Diffuse

*↑, increase; ↑↑, strong increase; ↓, decrease, ↓↓, strong decrease; ↔, negligible change.*

There are some limitations of our study; due to the special setting on the ward and the necessity of the longer EEG-recording as well as due to drop outs, the number of investigated subjects was rather low. What is more, though matched between groups, the span in age and epilepsy duration was rather high, which might have influenced our findings. Further studies in larger groups are thus necessary.

Using non–pharmaceutical interventions in epilepsy treatment often starts rather late in the course of treatment, especially since most of the current non–pharmaceutical interventions, such as epilepsy surgery or invasive stimulation methods, are accompanied by clearly defined risks. The non-invasive stimulation-based treatment is still in its infancy. The search for candidates who might profit from taVNS-based treatment should thus be extended, as it is common for epilepsy surgery and also ASM. Our experimental findings suggest, to our knowledge for the first time, different stimulation-mediated modifications of functional brain networks in different epilepsy types and point at potentially different responses of epileptic brain networks to taVNS in focal and generalized epilepsies. Further studies that investigate possible relationships between taVNS-induced modifications of functional brain networks and clinical efficacy are necessary to translate these experimental findings into clinical decision-making. The search for predictors of successful vagus nerve stimulation is a major challenge, for which first interesting insights have already been presented for iVNS (Workewych et al., 2020), but it is of importance to proceed and to install standardized protocols for experimental VNS research (Farmer et al., 2021) and also for future clinical applications.

## Data Availability Statement

The datasets presented in this article are not readily available because they contain information that could comprise the privacy of the participants. Requests to access the datastes should be directed to RW, randi.von.wrede@ukbonn.de.

## Ethics Statement

The studies involving human participants were reviewed and approved by the Ethics Committee of the University of Bonn. The patients/participants provided their written informed consent to participate in this study.

## Author Contributions

RW, CH, and KL: conceptualization. RW and KL: methodology, writing—original draft preparation, writing—review, editing, and supervision. RW, TR, TB, JP, SS, CH, and KL: validation, formal analysis, and data curation. All authors contributed to the article and approved the submitted version.

## Conflict of Interest

RW received once a fee for lecture from Cerbomed in 2016. The remaining authors declare that the research was conducted in the absence of any commercial or financial relationships that could be construed as a potential conflict of interest.

## Publisher’s Note

All claims expressed in this article are solely those of the authors and do not necessarily represent those of their affiliated organizations, or those of the publisher, the editors and the reviewers. Any product that may be evaluated in this article, or claim that may be made by its manufacturer, is not guaranteed or endorsed by the publisher.
