# Supplementary material for: Transcutaneous Auricular Vagus Nerve Stimulation Differently Modifies Functional Brain Networks of Subjects With Different Epilepsy Types
Source: Front Hum Neurosci. 2022 Jun 23;16:867563. doi: 10.3389/fnhum.2022.867563 (PMC9260140; doi:10.3389/fnhum.2022.867563)
Supplement: Supplementary file 1 [file Data_Sheet_1.PDF]

Supporting information

Appendix A: Details of neuropsychological assessment

Attention and executive functions
The EpiTrack® 3rd edition (Helmstaedter, 2019) is a screening tool which can be completed in 15 minutes. It consists of six subtests (response inhibition, visuo-motor speed, mental flexibility, visuo-motor planning, verbal fluency, and verbal working memory) resulting in a total score (age-corrected). The maximum score is 49 points; a total score in the range of 29 to 31 is rated as mild impairment, the cut-off score for severe impairment is ≤ 28 points. A gain of ≥ four points or the loss of ≥ three points between two assessments is rated as a significant intraindividual change. Parallel test versions were applied for reducing potential practice effects.

Subjective measures
For the assessment of self-perceived changes in different health domains all subjects answered a modified version of the Adverse Event Profile before and after stimulation. Subjects rated the presence and severity of the self-perceived changes on a four-tiered scale ranging from very good (0) to very bad (3) in three domains: (1) cognition (vigilance, energy, psychomotor speed, attention/ability to concentrate, fluent speech, verbal comprehension, word finding, remote memory), (2) behaviour (depression, anxiety, aggression, restlessness), (3) physiological symptoms (dizziness, drowsiness, nervousness, tremor, headache, nausea, dermatological symptoms, vision problems/double vision).

Questionnaire on the evaluation of the device
Seven ordinal questions were asked concerning handling, possibility to continue activities while using the device, feeling while using the device, comfort, suitability for long-term and repeated use.

Appendix B: Evolving Networks

We used a sliding-window approach (Kuhnert et al., 2010; Rings et al., 2019) to calculate the synchronization index r_{ij} (Mormann et al., 2000) between phase time series (derived adaptively from EEG data with the Hilbert transform, (Osterhage et al., 2007)) from all pairs of brain regions (i,j) sampled by the EEG electrodes. Non-overlapping windows had a duration of 20 s (5120 data points). The synchronisation index serves as an indicator for the strength of functional interactions in the epileptic brain network (Dickten et al., 2016) and is confined to the unit interval: $r_{ij} = 1$ indicates fully phase-synchronised brain regions and $r_{ij} = 0$ indexes no phase synchronisation. For subsequent analyses, we associated the sampled brain regions with network vertices and the calculated phase synchronisation indices between any pair of vertices with the weight of network edges. This resulted in a time-dependent sequence of weighted and fully connected brain networks.

Appendix C: Network characteristics

We assessed relevant global and local characteristics for each network that we derived from the above mentioned analysis of the three-hour EEG recording. In order to avoid possible transient effects, data from the first and last 15 min of each phase (one hour pre-stimulation, one hour taVNS stimulation, one hour post-stimulation) were excluded from the analysis.

In order to assess the global network scale, we estimated the topological characteristics average shortest path length L and average clustering coefficient C. The average shortest path length L is defined as the average number of steps along the shortest paths for all possible pairs of network vertices and, therefore, reflects the network's functional integration. For our weighted networks, we defined the `length' of a path between a pair of vertices as the inverse of the weight of the edge that connects the vertices (cf. (Lehnertz et al., 2014)), and used an algorithm proposed by Dijkstra (Dijkstra, 1959) to compute L. The clustering coefficient C is a measure of the degree to which vertices in a network tend to cluster together and, therefore, reflects the network's segregation. We made use of a definition of the clustering coefficient in weighted networks (Onnela et al., 2005) and calculated the average clustering coefficient C as the mean of clustering coefficients computed for all vertices.
In order to rate a network's stability and robustness, we estimated synchronisability S and assortativity A. *S*ynchronisability S assesses the stability of the network's synchronised state (Pecora and Carroll, 1998; Barahona and Pecora, 2002); it reflects the vulnerability/propensity of the synchronised state to get perturbed by some intervention. We computed S from the ratio of the largest and the smallest non-vanishing eigenvalue that we calculated for the network's Laplacian (Atay et al., 2006). Network's robustness, as assessed with assortativity A, was estimated by computing the Pearson correlation coefficient between the degrees of vertices at both ends of an edge (Newman, 2003; Bialonski and Lehnertz, 2013). To this end, we derived a connected binary network from the weighted network by thresholding, thereby requiring a constant edge density. A is confined to the interval [-1, 1] by definition, with positive/negative values indicating an assortative/disassortative network.

In order to assess the local network scale, we estimated the importance of vertices and edges employing two different and opposing centrality concepts that provide non-redundant information about the centrality of vertices and edges (Bröhl and Lehnertz, 2020; Geier and Lehnertz, 2017; Kuhnert et al., 2012; Bröhl and Lehnertz, 2019). As path-based centrality index, we utilized betweenness centrality C^B, which is defined as the ratio of all the shortest paths (between all pairs of vertices/edges) passing through a given vertex/edge to the sum of all shortest paths. As interaction-strength-based centrality index, we employed eigenvector centrality C^E of vertex/edge k which is defined as the k-th entry of the eigenvector corresponding to the dominant eigenvalue of the vertex/edge adjacency matrix (Bröhl and Lehnertz, 2019). A vertex/edge is important if the vertices/edges connected to it are also important.

References

Atay, F. M., Bıyıkoğlu, T., and Jost, J. (2006). Network synchronization: Spectral versus statistical properties. *Phys. D Nonlinear Phenom.* 224, 35–41. doi:https://doi.org/10.1016/j.physd.2006.09.018.

Barahona, M., and Pecora, L. M. (2002). Synchronization in small-world systems. *Phys. Rev. Lett.* 89, 54101. doi:10.1103/PhysRevLett.89.054101.

Bialonski, S., and Lehnertz, K. (2013). Assortative mixing in functional brain networks during epileptic seizures. *Chaos* 23, 33139. doi:10.1063/1.4821915.

Bröhl, T., and Lehnertz, K. (2019). Centrality-based identification of important edges in complex networks. *Chaos* 29, 33115. doi:10.1063/1.5081098.

Bröhl, T., and Lehnertz, K. (2020). Identifying edges that facilitate the generation of extreme events in networked dynamical systems. *Chaos* 30, 73113. doi:10.1063/5.0002743.

Dickten, H., Porz, S., Elger, C. E., and Lehnertz, K. (2016). Weighted and directed interactions in evolving large-scale epileptic brain networks. *Sci. Rep.* 6, 34824. doi:10.1038/srep34824.

Dijkstra, E. W. (1959). A note on two problems in connexion with graphs. *Numer. Math.* 1, 269–271. doi:10.1007/BF01386390.

Geier, C., and Lehnertz, K. (2017). Long-term variability of importance of brain regions in evolving epileptic brain networks. *Chaos* 27, 43112. doi:10.1063/1.4979796.

Helmstaedter, C. (2019). EpiTrack® – Veränderungssensitives kognitives Screening zur Beurteilung der Aufmerksamkeit und der Exekutivfunktionen für die Qualitäts- und Ergebniskontrolle der Behandlung von Patienten mit Epilepsie. *Eisai GmbH, Frankfurt am Main*.

Kuhnert, M.-T., Elger, C. E., and Lehnertz, K. (2010). Long-term variability of global statistical properties of epileptic brain networks. *Chaos* 20, 43126. doi:10.1063/1.3504998.

Kuhnert, M.-T., Geier, C., Elger, C. E., and Lehnertz, K. (2012). Identifying important nodes in weighted functional brain networks: a comparison of different centrality approaches. *Chaos* 22, 23142. doi:10.1063/1.4729185.

Lehnertz, K., Ansmann, G., Bialonski, S., Dickten, H., Geier, C., and Porz, S. (2014). Evolving networks in the human epileptic brain. *Phys. D Nonlinear Phenom.* 267, 7–15. doi:https://doi.org/10.1016/j.physd.2013.06.009.

Mormann, F., Lehnertz, K., David, P., and Elger, C. E. (2000). Mean phase coherence as a measure for phase synchronization and its application to the EEG of epilepsy patients. *Phys. D Nonlinear Phenom.* 144, 358–369. doi:https://doi.org/10.1016/S0167-2789(00)00087-7.

Newman, M. E. J. (2003). Mixing patterns in networks. *Phys. Rev. E. Stat. Nonlin. Soft Matter Phys.* 67, 26126. doi:10.1103/PhysRevE.67.026126.

Onnela, J.-P., Saramäki, J., Kertész, J., and Kaski, K. (2005). Intensity and coherence of motifs in weighted complex networks. *Phys. Rev. E. Stat. Nonlin. Soft Matter Phys.* 71, 65103. doi:10.1103/PhysRevE.71.065103.

Osterhage, H., Mormann, F., Staniek, M., and Lehnetz, K. (2007). Measuring Synchronization in the Epileptic Brain: a Comparison of Different Approaches. *Int. J. Bifurc. Chaos* 17, 3539–3544. doi:10.1142/S0218127407019330.

Pecora, L. M., and Carroll, T. L. (1998). Master Stability Function for Globally Synchronized Systems. *Phys Rev Lett* 80, 2109–2112. doi:10.1007/978-1-4614-7320-6_441-1.

Rings, T., von Wrede, R., and Lehnertz, K. (2019). Precursors of seizures due to specific spatial-temporal modifications of evolving large-scale epileptic brain networks. *Sci. Rep.* 9, 10623. doi:10.1038/s41598-019-47092-w.
